# The Plenum System of Ventilation

**Published:** 1909-05-15

**Authors:** 


### HOSPITAL ADMINISTRATION.

### CONSTRUCTION AND ECONOMICS.

### THE PLENUM SYSTEM OF VENTILATION.

A PROPOSED METHOD FOR ITS IMPROVEMENT.

A Means of Supplying Oxygen.

In an article contributed by the present writer to the columns of The Hospital of May 5, 1906, he pointed out that the cause of the almost universal complaint against the Plenum system is that something appears to be wanting in the air after it has been treated and delivered to the wards.

What is wanting is a certain proportion of oxygen. It was explained that as a result of the higher temperature usually maintained in wards and elsewhere, the quantity of oxygen passing through the ward in the ventilating air-current is less than it would be under what are known as natural conditions.

In one of the articles on the "Fitting and Furnishing of Wards and Operating Theatres" which appeared in The Hospital of November 17, 1906, was mentioned the fact that complaints arise in operating theatres that the atmosphere becomes very warm and somewhat enervating after operations have been going on for a certain time. In the following article the writer proposes a remedy for both of these complaints. The obvious remedy for a shortage of oxygen is the addition of oxygen. The oxygen may be delivered to the ventilating air-current as oxygen pure and simple or as ozone. If it is to be supplied as oxygen, the arrangement should be a very simple one. An iron bottle of compressed oxygen, the bottle having what engineers call a reducing-valve at its mouth, a valve which enables the egress of the oxygen to be controlled at will, would be placed in any convenient position near the entrance to the duct carrying air to the operating theatre or the ward, and a pipe from the bottle would pass into the duct through an air-tight gland, and would deliver the oxygen into the air-current in the form of a spray.

The Method of Mixing.

It would be a matter of some importance that the oxygen added to the air should be thoroughly mixed with it. That is to say, every cubic inch or fraction of a cubic inch of air should have its proportion of oxygen, and not that the oxygen should pass into the operating theatre in a separate stream. The spraying would be on somewhat similar lines to those adopted in the carburettor of a petrol engine, with the addition, if practicable, of a whirling motion given to the issuing oxygen, which would probably aid the thorough mixing with the air.

For operating theatres the quantity of oxygen delivered to the air-current would be controlled by an electric switch in the theatre, very similar to those employed for starting electric motors, the contacts being marked in percentages of oxygen. The switch in the operating theatres would control an electro-magnet of the solenoid type, which would open or close the valve on the oxygen bottle in accordance with the movements of the switch. In this manner, it would be very simple for one of the nurses to turn on more oxygen, or less, as instructed by the surgeon. The supply might also be controlled indirectly by an electric signalling arrangement between the operating theatre and the neighbourhood of the oxygen bottle, where an attendant would open or close the valve, in accordance with the signals received.

The same arrangement could be applied to wards and to any individual duct issuing into a portion of the ward, the oxygen bottle being fixed at the point where that particular duct joined with the main supply.

Where ozone is employed, what is termed an ozonising apparatus would be used.

Ozonisation.

Ozone is formed by the oxidising of a small portion of the oxygen of the air whenever air passes across a space where a high-tension electrical discharge is taking place. There are several ozonising apparatus on the market, all constructed on very much the same lines. There is what is called an electrical condenser, arranged to be constantly charged and discharged from an alternating-current service at a high tension; and there is a fan, driven by an electric motor, causing air to pass through the space where the electrical discharge is taking place. The apparatus usually includes an electrical transformer, and where the electrical service is on the continuous-current system, it also includes an apparatus for converting it to alternating current. The ozonising apparatus may be placed directly in the air-current, with a by-pass allowing all, or a portion, of the air passing into the duct to avoid the ozoniser; or, as the writer would rather prefer, a separate ozonised air-current may be delivered to the air in the duct, in a similar manner to that described with oxygen. Where the whole of the air in the duct is ozonised, the by-pass would be provided with a valve controlled from the operating theatre or the ward, which would be opened or closed in accordance with the requirements, by means of an electric service, just as with the oxygen bottle; or it might be controlled by signal, an attendant opening and closing the valve as the signals ordered. Where a separate ozonised air-current was delivered to the duct, the quantity of ozonising air delivered could be controlled either by varying the speed of the fan—this being controlled from the operating theatre or the ward—or by controlling the ingress of the ozonised air to the duct by a valve similar to that described for the by-pass.

Practical Points.

The whole arrangement should be exceedingly simple, though certain details would have to be worked out. The quantities of either oxygen or ozonised air dealt with would be very small, and the apparatus designed to control them would also be small, and would not absorb much power. The quantity of oxygen converted into ozone by the ozonising apparatus is very small indeed. It has been given to the writer as something like three parts of ozone in one million of air. The quantity necessary under different conditions would be partly a matter of calculation and partly of experiment. Two points should be noted: the compressed oxygen issuing into the air-duct would tend to cool the air as it expanded; but this could easily be provided for, and as the quantity of oxygen delivered would be small, the cooling effect would also be small.

Both arrangements could be adapted, with a little working out, for those cases where wards, etc., are ventilated and warmed by means of radiators placed in front of gratings in the outer walls. Small bottles of oxygen, or small ozonising apparatus, could easily be fixed in such a position that a small quantity of oxygen, or of oxonised air, could mingle with the warmed air-current passing into the ward. One form of apparatus has already been adapted for ozonising the air employed in cooling the "wort" in a brewery. The hot liquor produced by boiling the malt in water, from which beer is afterwards made—technically known as "wort"—has to be cooled before it is taken to the fermenting tun. The cooling is done very largely by evaporation, and it has been thought that, by ozonising the air employed in cooling, any germs present that may have been drawn in from the outside atmosphere would be destroyed.

The plan suggested by some of the makers of ozonising apparatus, of putting the apparatus down anywhere in a ward, and allowing it to deliver ozone to the atmosphere of the ward, can hardly be recommended. As the writer understands, before all things it is necessary, in ventilation, that the whole of the air of the ward or operating-theatre—more particularly the latter—should be dealt with.

